# Personal psychedelic experience of psychedelic therapists during training: should it be required, optional, or prohibited?

**DOI:** 10.1080/09540261.2024.2357669

**Published:** 2024-06-12

**Authors:** Daniel Villiger

**Affiliations:** Uehiro Centre for Practical Ethics, University of Oxford, Oxford, UK

**Keywords:** Psychedelic-assisted therapy, personal psychedelic experience, psychedelic ethics, training, personal therapy

## Abstract

Personal psychedelic experience is common among psychedelic therapists and often considered to be a necessary aspect of training: only personal psychedelic experience allows psychedelic therapists to properly guide patients through their own psychedelic experience, to truly understand that experience, and to help them integrate it into their lives. But is this really true? The present paper examines the value of therapists’ personal psychedelic experience, why this value may be higher than that of personal experience with other psychotropic drugs, and whether it justifies a requirement of personal psychedelic experience for psychedelic therapists. The analysis, which also considers the literature on therapists’ personal experience with the mental disorder being treated or with psychotherapy, concludes that the current evidence does not justify making personal psychedelic(-like) experience a requirement for psychedelic therapists. However, because therapists’ personal psychedelic experience can be valuable to both therapists and patients, and because the likelihood of harm is very low, psychedelic therapists should be given the opportunity to have a psychedelic experience during their training.

## Introduction

Psychedelics have made an impressive comeback in mental health care. In the last decade, an increasing number of studies have investigated the therapeutic effects of classic serotonergic psychedelics, including lysergic acid diethylamide (LSD), psilocybin, and N,N-dimethyltryptamine (DMT).^1^ The present results are promising and suggest efficacy in the treatment of major depressive disorder (Carhart-Harris, Bolstridge, et al., 2016; Carhart-Harris et al., 2018, 2021; Goodwin et al., 2022; Palhano-Fontes et al., 2019; Raison et al., 2023; Reiff et al., 2020; Rotz et al., 2023; Sanches et al., 2016), anxiety with and without a life-threatening illness (Gasser et al., 2014; Griffiths et al., 2016; Holze et al., 2023; Johnson & Griffiths, 2017; Reiff et al., 2020; Ross et al., 2016), and alcohol and smoking cessation (Bogenschutz et al., 2015; Garcia-Romeu et al., 2019; Johnson, Garcia-Romeu, & Griffiths, 2017; Johnson, Garcia-Romeu, Johnson, et al., 2017; Krebs & Johansen, 2012). Meta-analyses and systematic reviews underscore the potential benefits of psychedelics in mental health care and suggest that psychedelics can be safely used in the clinical context (Andersen et al., 2021; Bender & Hellerstein, 2022; Haikazian et al., 2023; Ko et al., 2023; Maia et al., 2022; Romeo et al., 2020; Roscoe & Lozy, 2022; Simonsson et al., 2023; Wheeler & Dyer, 2020).

Among the classic serotonergic psychedelics, psilocybin is currently receiving the most attention. In February 2024, there were 84 ongoing clinical studies using psilocybin, including four phase 3 trials (ClinicalTrials, 2024). For DMT and LSD, there were seven and four ongoing clinical trials, respectively. In addition to their use in clinical trials, psychedelics have begun to be used clinically in non-research contexts too. Countries such as Canada, Switzerland, and Israel grant psychiatrists special permission to use psychedelics under certain conditions, and in 2023 Australia has become the first country to generally allow psilocybin for treatment (Ducharme, 2023; Haridy, 2023). Taken together, we can already observe significant clinical use of psychedelics, and it is very likely that this use will continue to increase in the coming years.

A unique feature of the clinical use of psychedelics compared to other psychotropic drugs is that psychedelics are not simply prescribed to patients to take on their own. Instead, patients take psychedelics in a guided session that is embedded in one or more preparation sessions before and one or more integration sessions after the psychedelic session. Because of the therapeutic embedding of psychedelics, the overall treatment is called *psychedelic-assisted therapy* (PAT), emphasizing its inherent combination of pharmacotherapy with psychotherapy.^2^ This unique feature of psychedelics also makes the role of psychedelic therapists unique: they prescribe a drug like a psychiatrist, but at the same time they also guide the patient through the psychedelic experience; and they perform a form of psychotherapy like a psychotherapist, but with an emphasis on the psychedelic experience.

The unique role of psychedelic therapists has led to lively debates about what training they should have, and in particular whether they should have personal psychedelic experience themselves (Earleywine et al., 2023; Emmerich & Humphries, 2023; Nielson & Guss, 2018). Traditionally, there has been agreement on the essential need for psychedelic therapists to have personal experience with the psychedelic substance they administer (Grof, 2005; Hoffer & Osmond, 1967; Metzner, 2015; Phelps, 2017; Sherwood et al., 1962). This need has recently be questioned (Emmerich & Humphries, 2023; Hendricks & Nichols, 2023; Tai et al., 2021). The present paper examines the value of psychedelic therapists’ personal psychedelic experiences and analyzes whether this value justifies a requirement for such experiences. In doing so, the paper also draws on the literature on therapists’ personal experience with the mental disorder being treated or with psychotherapy. The analysis concludes that while psychedelic therapists should be given the opportunity to have a psychedelic experience during their training, current evidence does not justify making a personal psychedelic(-like) experience a requirement for psychedelic therapists.

## The value of therapists’ personal experience

A therapist’s personal experience with the treatment of a mental disorder can come in three types: personal experience with the mental disorder, personal experience with psychotherapy, and personal experience with the prescribed psychotropic drug. A psychedelic therapist who has at least once taken psychedelics is an example of the latter. But before we analyze this in more detail, let us briefly discuss the first two types of personal experience.

### Personal experience with the mental disorder being treated

Obviously, having personal experience with the mental disorder being treated is neither a requirement nor a recommendation for therapists. Nevertheless, the advantages and disadvantages of therapists’ personal experience with mental disorders have long been debated in the literature. On the one hand, personal experience with mental disorder gives therapists some form of experiential knowledge that cannot be gained otherwise. In other words, without their personal experience of the mental disorder, knowing what it is like phenomenologically to suffer and recover from or learn to cope with that disorder would remain epistemically inaccessible. Having access to such knowledge can help therapists to better understand and empathize with patients (Elliott & Ragsdale, 2020). For example, in a qualitative study of eating disorder therapists with a personal history of eating disorder, almost all participants said that their personal history helped them develop a greater understanding of the disorder, increased empathy, and a more positive outlook (Warren et al., 2013). Another study on the topic finds that recovered therapists can use their experiential knowledge ‘as a therapeutic intervention with specific goals, such as providing the patient with insight into the recovery process, establishing a working relationship, and enhancing hope for recovery’ (de Vos et al., 2016, p. 207). In addition, almost all patients that were part of this study reported that being treated by a therapist with a personal history of eating disorder had a positive impact on their recovery process.

On the other hand, personal experience with a mental disorder can also be problematic when treating that mental disorder. First, therapists may project themselves onto patients, with the risk of neglecting that the patient’s mental disorder may manifest differently from their own, and thus their experiential knowledge may not be fully applicable to the patient (de Vos et al., 2016). For example, Hecksher (2007) argues that similar personal histories among substance abuse counselors and clients may interfere with counselor objectivity in assessment and treatment planning. Specifically, there is a risk that the counselor will impose their standards or criteria for success on the client without evaluating the actual complaints. Second, therapists may become overinvolved with patients, losing their professional distance to patients and getting triggered themselves (Warren et al., 2013).

There is mixed support that a therapist’s personal experience with a mental disorder has positive effects on treatment outcomes. There is evidence showing that self-disclosure increases therapeutic effects (Barrett & Berman, 2001). Furthermore, disclosures that convey similarity between therapist and client seem to be more effective than disclosures that convey neither similarity nor dissimilarity (Levitt et al., 2015). This suggests that therapists increase therapeutic effects when they have a personal history of mental disorder and disclose it. But then again, a meta-analysis that compares treatment outcomes of substance abuse counselors with and without a personal history of substance abuse found no significant differences (Culbreth, 2000). Still, it is important to note that there is no evidence showing that a therapist’s personal experience with a mental disorder has a negative effect on treatment outcomes.

### Personal experience with psychotherapy

It is recommended and often required for psychotherapists to have personal experience with psychotherapy. For example, in many European countries, the requirement for 40 or more personal therapy sessions during psychotherapy training is common (Malikiosi-Loizos, 2013). While personal therapy can be a source of personal development, it is also believed to promote professional development in two ways. (1) It adds knowledge and skills that help psychotherapists understand the therapeutic process more effectively. Being the patient for once in the therapist-patient interaction provides the therapist with a different perspective on the therapeutic setting and the therapeutic process. In other words, it provides them with (some) experiential knowledge of what it is like to attend psychotherapy as a patient. This sensitizes them to the thoughts, feelings, needs, and expectations a patient may experience during the therapeutic process, fostering understanding and empathy for the patient; gives them a better understanding of the therapist-patient dynamic and their interpersonal relationship; and allows them to observe clinical tools. (2) Personal therapy increases self-awareness, improving the therapist’s mental and emotional functioning. A high degree of self-awareness is important because the therapist must be able to identify countertransference reactions occurring as a result of the patient’s transference and reactions that are projections of the therapist’s own unresolved issues. In addition, it ensures that the therapist is not completely overwhelmed by seeing patients who are often in extreme distress (Kumari, 2011).

Self-report studies show that a large majority of therapists find attending personal therapy helpful (Moe & Thimm, 2021). Specifically, therapists reported increased knowledge of the therapist-patient relationship and therapeutic dynamics. Moreover, they stated that personal therapy gave them additional insight into how essential qualities such as empathy, warmth, transference, countertransference, tolerance, and patience are in psychotherapy. However, there is also a small proportion of therapists, about 1% to 10% according to Orlinsky et al. (2005), who describe their personal therapy as harmful and not helpful.

Despite the self-reported benefits of personal therapy for therapists’ professional development, there is no evidence that personal therapy leads to better treatment outcomes (Moe & Thimm, 2021): Most studies find no differences in treatment outcomes between therapists who have attended personal therapy and those who have not. Yet, it must be noted that the studies examining this issue have methodological weaknesses, such as small sample sizes and inadequate controls. Therefore, current research does not allow for a definitive conclusion regarding the impact of personal therapy on treatment outcomes.

### Personal experience with the administered psychedelic substance

There is no psychotropic drug that therapists are encouraged, let alone required, to take as a form of self-experience during training. And while there is anecdotal evidence of psychiatrists trying psychotropic drugs themselves for scientific reasons or simply out of curiosity, this is not the norm (Wachter et al., 2021). Psychedelics seem to be an exception in this regard. It has been repeatedly argued that psychedelic therapists need to have personal experience with the psychedelic substance they administer (cf. Phelps, 2017). In line with that, a survey on psychedelic therapists associated with the Usona Institute’s Phase II clinical trial found that of the 22% who completed the questionnaire, 88% reported having taken a psychedelic at least once (Aday et al., 2023).

The reason for the special status of psychedelics among psychotropic drugs lies in the assumed therapeutic mechanisms of PAT. In normal pharmacotherapy, the psychotropic drug itself should enable the patient to regain a functional state with reduced or, at best, resolved symptoms. Ideally, the experience of taking the psychotropic drug involves only the slow to rapid transition to the (more) functional state, with other experiential changes being unwanted side effects. Therefore, taking the ideal psychotropic drug should not result in gaining much experiential knowledge, especially if one is already familiar with the functional state the drug is trying to induce.

Psychedelics differ from a normal psychotropic drug in four important ways: (1) Psychedelics lead to profound changes in mood and perception, including near-death-like experiences (Timmermann et al., 2018), ego dissolution (Nour et al., 2016), altered time perception (Wackermann et al., 2008), paranoid and delusional thinking (Carhart-Harris, Kaelen, et al., 2016), and more. That is why a psychedelic experience is often described as ineffable, implying that one cannot conceptualize the manifestation of a first-time psychedelic experience (cf. Villiger, 2024). Taking psychedelics for the first time therefore leads to a considerable gain in experiential knowledge that seems otherwise unattainable. (2) The current body of evidence suggest that the phenomenological effects of psychedelics are not just an unnecessary side-effect but constitutive for therapeutic effects: even when the rated intensity of the overall drug effects is statistically controlled for, the more mystical patients rate their psychedelic experience, the greater the therapeutic effects (Yaden & Griffiths, 2020). (3) The setting in which one takes psychedelics has a great impact on how the psychedelic experience unfolds (Hartogsohn, 2020). Since therapists are part of the setting, they can positively or negatively affect the patient’s psychedelic experience. For example, it is common for therapists to comfort patients who feel distressed during their psychedelic experience by holding their hand (cf. Smith & Sisti, 2021).^3^ (4) A psychedelic session is followed by one or more integration sessions where therapists support patients in translating their psychedelic experience into sustainable real-life changes. In fact, it has been hypothesized that the actual acquisition and integration of new insights occurs after the psychedelic experience (Carhart-Harris & Friston, 2019). Thus, via integration sessions, therapists play an important role in what patients take away from their psychedelic experience. For example, in a study on therapists who tried psychedelics for the first time, one participant wrote: ‘My second session has in retrospect been a bust; the guide resisted my attempts to make it relevant to my life’ (Nielson, 2021, p. 8). The same participant later noted that his experience reminded him of ‘how good the guide has to be’ (p. 9).

These four differences form the basis for the argument that psychedelic therapists must have personal psychedelic experience: Not only the neurobiological effects of the administered psychedelic but also the subjective experience it induces is important for therapeutic effects. That is why PAT therapists are not simply psychedelic supervisors who monitor the patient’s physical activity but psychedelic guides who try to facilitate a positive/therapeutic psychedelic experience. And just as travel guides must be familiar with the terrain through which they lead, psychedelic therapists must be familiar with the mental states a patient is experiencing: During a psychedelic trip, therapists need to be familiar with psychedelic mental states because only then can they guide the patient’s experience in a positive direction. As O’Sullivan, one of the health professionals granted exemptions from Canada’s drug laws to use psilocybin, said in an interview: ‘[I]t is absolutely imperative that the therapist have familiarity with the realms of the human unconscious that are visited under psychedelics because they can help guide the patient through situations that might seem utterly bizarre, even psychotic to an untrained therapist’ (Dubinski, 2020). After the psychedelic trip, therapists need to be familiar with psychedelic mental states because only then can they help patients properly interpret their psychedelic experience and translate it into sustainable real-life changes. As Hofmann (2005) wrote: ‘[Personal psychedelic experience would] provide the doctors with direct insight, based on first-hand experience into the strange world of LSD inebriation, and make it possible for them to truly understand these phenomena in their patients, to interpret them properly, and to take full advantage of them’ (p. 76). Finally, since the phenomenology of a psychedelic experience appears to be epistemically accessible only by undergoing such an experience, being familiar with psychedelic mental states requires personal psychedelic experience.

While this line of argument seems prima facie convincing, it can be attacked on two grounds. (1) Familiarity with psychedelic-like mental states may not necessitate personal experience with psychedelics. Emmerich and Humphries (2023) argue that if the experiential knowledge to which a psychedelic experience leads lies in the affective state it induces and the profound truths or meanings it makes one realize, then other experiences can also provide some of this knowledge. This includes near-death experiences, non-drug-related forms of mystical or religious experiences, holotropic breathwork, and the kinds of states achieved during advanced meditation or induced by sensory deprivation. Importantly, by changing the training requirement to familiarity with *psychedelic-like* mental states, individuals who are medically contraindicated to taking psychedelics (e.g. because of a personal or family history of mental disorder) are no longer excluded from becoming psychedelic therapists. On this basis, Emmerich and Humphries reject the requirement that psychedelic therapists have drug-induced psychedelic experiences. However, the authors remain somewhat ambiguous about whether (some) experiential knowledge of psychedelic-like mental states is still necessary for psychedelic therapists, which brings us to the next objection. (2) There is no evidence that psychedelic therapists with psychedelic experience or experience of psychedelic-like mental states are better than those without. This is because no study has ever examined the effect of the psychedelic therapist’s personal psychedelic(-like) experience on treatment outcomes. There are only self-reports by psychedelic therapists that they have benefited from their own psychedelic experiences, both professionally and personally (cf. Nielson, 2021). Whether this translates into better treatment outcomes remains to be tested.

In fact, the research discussed above on the effects of therapists’ personal experience with the mental disorder being treated or with psychotherapy casts doubt on whether personal psychedelic experience improves treatment outcomes. Therapists with a personal history of the mental disorder being treated have access to experiential knowledge that other therapists do not have and say that such access helps them better understand and empathize with their patients. Nevertheless, there is no conclusive evidence that this leads to better treatment outcomes. Why should that be different for psychedelic therapists who say that their personal psychedelic experience helps them better understand and empathize with their patients? Similarly, most psychotherapists say that attending personal therapy is or has been helpful, both professionally and personally, as it gave them additional insight into the therapist-patient relationship, the therapeutic process, and their own issues. Nevertheless, there is no conclusive evidence that personal therapy leads to better treatment outcomes. Why should that be different for psychedelic therapists who say that their personal psychedelic experience helps them better understand therapist-patient dynamics and the therapeutic process of PAT? Ultimately, a psychedelic experience in a training setting may differ significantly from a psychedelic experience in a therapeutic setting because of their different purposes, raising the question of whether the former truly provides epistemic access to the latter. Taken together, the burden of proof is clearly on those who claim that the therapist’s personal psychedelic experience improves treatment outcomes.^4^

Only if such evidence can be provided may a requirement of personal psychedelic(-like) experience for psychedelic therapists be justified. If we follow principlism (cf. Beauchamp & Childress, 2013), there are two ways to do this: If there is evidence that therapists’ personal psychedelic(-like) experiences benefit patients by improving therapeutic effects, then the requirement can be justified under the duty of beneficence. If there is evidence that therapists’ personal psychedelic(-like) experiences reduce negative effects (e.g. fewer serious adverse events), then the requirement can be justified under the duty of non-maleficence.

While a requirement that psychedelic therapists have psychedelic(-like) experiences is not currently justifiable, there are still reasons to have a psychedelic experience as a psychedelic therapist. As noted above, self-reports by psychedelic therapists suggest that personal psychedelic experiences have had positive effects on them, both professionally and personally. In this context, it is important to note that the safety of administering psychedelics to healthy people has been demonstrated in several studies (cf. Yaden et al., 2022), and there are no indications that having a psychedelic experience as part of the therapeutic training increases the willingness to take psychedelics again outside of a medical setting (Nielson, 2021).

Finally, a survey of depressed patients found that, on average, personal psychedelic experience among psychedelic therapists was rated as at least ‘somewhat important’ (Earleywine et al., 2023). Thus, many patients are likely to prefer a psychedelic therapist with personal psychedelic experience to one without. This may be because the therapist’s personal experience with psychedelics increases patients’ trust in the therapist’s reassurances and ability to empathize with them and their situation (cf. Earleywine et al., 2023; Engel et al., 2022). Ultimately, such an increase in trust may even lead to better treatment outcomes, for example via improving the therapeutic alliance (cf. Levin et al., 2024) – a hypothesis that remains to be tested. However, self-disclosure of personal psychedelic experience also presents potential challenges: First, by inducing expectations and initiating comparisons, the therapist’s account of their own psychedelic experience may affect the unfolding and interpretation of the patient’s psychedelic experience. In addition, poor use of self-disclosure may inappropriately alter the dynamics of the therapist-patient relationship (cf. Brennan et al., 2021). Thus, self-disclosure of personal psychedelic experience is a delicate task that needs to be done skillfully and that is worthy of being explored in more detail in future research. Second, a survey found that psychedelic researchers with personal psychedelic experience are seen as more biased, less professional, and less honest than psychedelic researcher without such experience (Forstmann & Sagioglou, 2021). Could the same be true for psychedelic therapists? Probably not, because personal psychedelic use is mainly accused of compromising scientific objectivity, and while scientific objectivity is essential in psychedelic research, it is less so in psychedelic practice (cf. Kious et al., 2023). Besides, if the same were true for psychedelic therapist, it would be difficult to explain why many patients are likely to prefer a psychedelic therapist with personal psychedelic experience to one without.

Taken together, therapists’ personal psychedelic experiences can be valuable to both therapists and patients, and the likelihood of harm is very low. Because of that, it seems reasonable to give psychedelic therapists a legal opportunity to have a psychedelic experience during their training (with the same inclusion criteria as psychedelic studies with healthy participants).

## Conclusion

This paper analyzed whether personal psychedelic experience of psychedelic therapists during training should be required, optional, or prohibited. The analysis found no evidence demonstrating that a therapist’s personal psychedelic(-like) experience improves therapeutic effects or reduces negative effects. Furthermore, it found no conclusive evidence that therapists’ personal experience with the mental disorder being treated or with psychotherapy improves treatment outcomes, suggesting that the same may be true for therapists’ personal psychedelic(-like) experience. Therefore, a requirement of personal psychedelic(-like) experience for psychedelic therapists is not justified. However, as personal psychedelic experiences can be valuable and have a low risk profile, they should be offered to therapists during PAT training.

On a final note, while the line of argument presented in this paper refers to classic serotonergic psychedelics, it can also be applied to other kinds of psychoactive therapeutics in which the acute subjective effects may play some role (e.g. ketamine or MDMA): Only if there is evidence that personal experience with such a substance improves treatment outcomes can a requirement for such experience as a therapist be justified. At the same time, if the substance has a very low likelihood of harm and self-reports indicate that therapists’ personal experience with it is professionally valuable, trainees should be given the opportunity to try it.

## Notes

1 In addition, studies with 3,4-meth­y­lene­di­oxy­methamphet­amine (MDMA), which is sometimes also classified as a psychedelic substance, have shown ­treatment benefits for post-traumatic stress disorder (Mithoefer et al., 2018; Ot’alora G et al., 2018), including phase 3 trials (Mitchell et al., 2021, 2023), and for social anxiety among patients on the autism spectrum (Danforth et al., 2018). However, the focus of this paper will be on the classic serotonergic psychedelics.

2 Of course, pharmacotherapy and psychotherapy are also combined in other treatments (e.g. psychotherapy with antidepressants). However, the combination is usually not mandatory, and the two therapies are not that much intertwined.

3 Note that the method of therapeutic touch, which dates back to PAT practices in the 1960s and has long been regarded best practice in modern PAT studies, has come under scrutiny in recent years. Devenot et al. (2022) argue that research on the subject is insufficient to justify it on scientific grounds, considering that therapeutic touch can (unintentionally) cross boundaries.

4 For research examining this claim, it would also be interesting to analyze whether/how the three types of personal experiences (i.e. the mental disorder being treated, psychotherapy, and psychedelics) interact with each other in PAT.
